# Elevated Expression of MiR-17 in Microglia of Alzheimer’s Disease Patients Abrogates Autophagy-Mediated Amyloid-β Degradation

**DOI:** 10.3389/fimmu.2021.705581

**Published:** 2021-07-27

**Authors:** Shady Estfanous, Kylene P. Daily, Mostafa Eltobgy, Nicholas P. Deems, Midhun N. K. Anne, Kathrin Krause, Asmaa Badr, Kaitlin Hamilton, Cierra Carafice, Ahmad Hegazi, Arwa Abu Khweek, Hesham Kelani, Shahid Nimjee, Hamdy Awad, Xiaoli Zhang, Estelle Cormet-Boyaka, Hesham Haffez, Sameh Soror, Adel Mikhail, Gerard Nuovo, Ruth M. Barrientos, Mikhail A. Gavrilin, Amal O. Amer

**Affiliations:** ^1^Department of Microbial Infection and Immunity, Infectious Diseases Institute, The Heart and Lung Research Institute, Ohio State University, Columbus, OH, United States; ^2^Biochemistry and Molecular Biology Department, Faculty of Pharmacy, Helwan University, Cairo, Egypt; ^3^Institute for Behavioral Medicine Research, Ohio State University, Columbus, OH, United States; ^4^Max Planck Unit for the Science of Pathogens, Berlin, Germany; ^5^Department of Biology and Biochemistry, Birzeit University, West Bank, Palestine; ^6^Department of Anesthesiology, Wexner Medical Center, The Ohio State University, Columbus, OH, United States; ^7^Department of Neurological Surgery, Wexner Medical Center, The Ohio State University, Columbus, OH, United States; ^8^Center for Biostatistics, Ohio State University, Columbus, OH, United States; ^9^Department of Veterinary Biosciences, The Ohio State University, Columbus, OH, United States; ^10^Center of Excellence, Helwan Structure Biology Research, Cairo, Egypt; ^11^GNOME DIAGNOSTICS, Department of Scientific Research, Powell, OH, United States; ^12^Department of Internal Medicine, Ohio State University, Columbus, OH, United States

**Keywords:** autophagy, microglia, NBR1, microRNA, Amyloid-β, Alzheimer’s disease, Mir17-92a

## Abstract

Autophagy is a proposed route of amyloid-β (Aβ) clearance by microglia that is halted in Alzheimer’s Disease (AD), though mechanisms underlying this dysfunction remain elusive. Here, primary microglia from adult AD (5xFAD) mice were utilized to demonstrate that 5xFAD microglia fail to degrade Aβ and express low levels of autophagy cargo receptor NBR1. In 5xFAD mouse brains, we show for the first time that AD microglia express elevated levels of microRNA cluster Mirc1/Mir17-92a, which is known to downregulate autophagy proteins. By *in situ* hybridization in post-mortem AD human tissue sections, we observed that the Mirc1/Mir17-92a cluster member *miR-17* is also elevated in human AD microglia, specifically in the vicinity of Aβ deposits, compared to non-disease controls. We show that NBR1 expression is negatively correlated with expression of *miR-17* in human AD microglia via immunohistopathologic staining in human AD brain tissue sections. We demonstrate in healthy microglia that autophagy cargo receptor NBR1 is required for Aβ degradation. Inhibiting elevated *miR-17* in 5xFAD mouse microglia improves Aβ degradation, autophagy, and NBR1 puncta formation *in vitro* and improves NBR1 expression *in vivo*. These findings offer a mechanism behind dysfunctional autophagy in AD microglia which may be useful for therapeutic interventions aiming to improve autophagy function in AD.

## Introduction

Alzheimer’s disease (AD) is the most common form of dementia worldwide and is characterized by chronic and irreversible neuronal degradation downstream of complex pathophysiological processes (1). One hallmark of AD is the presence of plaques primarily composed of insoluble amyloid-β (Aβ). Aβ accumulates in AD in a variety of forms, including soluble, oligomeric and fibrillar (2). The rate of Aβ clearance in healthy individuals is comparable to the rate of production (3). Accumulation of Aβ in the brain is a predisposing factor contributing to AD pathobiology usually preceding Tau tangle pathology (4). This strongly suggests that improving Aβ clearance is needed at early stages of the disease. To do so, a better understanding of the underlying defect is urgently required.

Microglia, brain resident immune cells, maintain tissue homeostasis by phagocytosis of dead cells, debris and toxic material such as extracellular Aβ (5). Microglia can participate in Aβ clearance by releasing enzymes that degrade Aβ in the extracellular space or by phagocytosing Aβ and degrading Aβ intracellularly within lysosomal compartments (6–8). Despite the capacity of healthy microglia to clear different forms of Aβ, reports suggest that AD microglia do not efficiently perform this function throughout AD (8–10). One proposed mechanism of Aβ degradation by microglia after internalization is autophagy (11–13). Autophagy is a conserved homeostatic cellular process for the degradation of extracellular and intracellular materials (14). Typically, extracellular materials destined for autophagy are phagocytosed by immune cells and marked by “eat me” signals, including poly-ubiquitin (14). Then, autophagy receptors such as p62/SQSTM1, NDP52, OPTN (optineurin), or NBR1 (neighbor of BRCA1 gene 1) bind to the ubiquitinated cargo and recruit LC3, bringing the nascent autophagosome (14, 15). Cargo-carrying autophagosomes mature and fuse with lysosomes where degradation of the autophago-lysosome-enclosed material occurs (14). The implication of dysfunctional autophagy in AD pathogenesis and Aβ degradation have been investigated but studies mainly focused on neuronal cells (16, 17). Few studies have shown that dysfunctional autophagy has significant consequences in AD microglia, including failure to regulate harmful neuroinflammation (17, 18). Emerging reports described a role for autophagy-mediated degradation of Aβ in AD, yet these studies were performed in cell lines or neonatal microglia, but not in diseased adult AD microglia and underlying mechanisms for dysfunctional autophagy in AD were not offered (11–13). Prolonged exposure to Aβ has been shown to disrupt autophagy in microglia, however, underlying cellular changes in response to Aβ leading to this disruption also remain unknown (19).

MicroRNAs (*MiRs*) are implicated in autophagy regulation (20) and AD progression (21), though there is no evidence of *MiRs* regulating autophagy in AD microglia. *MiRs* are evolutionarily conserved noncoding RNAs which mediate post-transcriptional gene silencing by binding to the 3’-untranslated region (UTR) or open reading frame (ORF) region of target mRNAs (22). Considering the strong implication of *MiRs* in both AD pathogenesis (21, 23) and autophagy function (20) and given the lack of evidence linking these two fundamental processes, we searched for *MiRs* that regulate autophagy whose expression is altered in AD. Members of the Mirc1/Mir17-92 *MiR* cluster are known to target essential autophagy molecules (24). Diminished expression of an individual autophagy protein targeted by elevated *MiRs* can halt autophagic function which will not be recovered by expression of other autophagy proteins (24). The Mirc1/Mir17-92 cluster, represented by *miR-17-5p*, *miR-18a*, *miR-19a*, *miR-20a*, *miR-19b-1*, and *miR-92a-1*, is conserved among vertebrates and associated with roles in cell cycle, tumorigenesis, and aging (25).

In this study we investigate the role of the Mirc1/Mir17-92 cluster in AD. We find that expression of the cluster increases specifically in microglia of AD mouse model (5xFAD) mice as they age. This upregulation is more prominent in the brains of AD human patients, when comparing with non-AD age- and sex-matched counterparts. Notably, expression of *miR-17-5p* (*miR-17*) is elevated in microglia adjacent to Aβ deposits but not in microglia away from deposits in the same human brain tissue sample. In addition, increased expression of *miR-17* correlates with reduced expression of target autophagy cargo receptor NBR1, which we demonstrate is needed for efficient Aβ degradation. Further, elevation of cluster component *miR-17* is largely responsible for defective clearance of Aβ by AD microglia, as inhibiting *miR-17* alone improves degradation of Aβ, autophagy and NBR1 puncta formation *in vitro* and restores expression of NBR1 in microglia *in vivo*. Our study advances our understanding of the role of microglial autophagy in the clearance of Aβ and provides a mechanistic basis for dysfunctional autophagy in AD microglia in human and mice.

## Materials and Methods

### Human Samples From NIH Biobank

Human temporal lobe brain tissue sections (Brodmann area 38) from patients with AD and age-matched controls without dementia were obtained from the NIH biobank in accordance with our institution’s IRB. These snap-frozen tissue samples were used for all TRIzol homogenization, RT-qPCR and immunoblot analysis. Range of hours until freeze, age, and sex are in **Supplemental Table 1**. The presence of Aβ deposits within homogenized samples is shown in **Supplemental Figure 2B**. Equal number of samples (n=15) for AD and age- and sex-matched non-AD controls were analyzed in determining *MiR* expression (**Figure 5A**) and (n=10) total brain homogenate NBR1 and Aβ burden (**Supplementary Figure 2**). Results were similar when data analysis was segregated by sex (data not shown). AD (BRAAK III or IV) and age-matched control hippocampal tissue sections utilized for *in situ* hybridization (ISH) and immunohistochemical analysis were obtained from the Discovery Life Science (DLS) tissue bank by G. Nuovo (26). Analysis of NBR1 in different cell types was performed in 3 AD and 3 control samples (**Figure 3A**). Analysis of NBR1 in different AD brain regions was performed in Aβ-negative and Aβ-positive regions from 3 unique patient samples (**Figure 3C**). Analysis of *miR-17* and Aβ was performed in 4 AD and 4 control samples, or Aβ-negative and Aβ-positive regions from 4 unique patient samples (**Figures 5B–E**). Analysis of NBR1 with either TMEM (microglia) or neurons (pyruvate dehydrogenase) was performed in 3 unique AD patients in Aβ-positive regions (**Figures 5F, G**). Correlation analyses of *miR-17* and NBR1 in **Figure 7** were performed on 4 patients for AD and 4 patients for non-AD.

### Mice

C57BL/6 wild-type (WT) mice, 5xFAD mice and non-carrier controls (MMRRC stock #34840) were obtained from the Jackson Laboratory (Bar Harbor, ME, USA). The 5xFAD (AD) (B6SJL-Tg (APPSwFlLon, PSEN1* M146L* L286V) 6799Vas/Mmjax) mouse is a double transgenic APP/PS1 mouse model that co-expresses five AD mutations leading to accelerated plaque formation and increased Aβ42 production. This AD mouse model over-expresses APP with K670N/M671L (Swedish Mutation), I716V (Florida mutation), and V717I (London mutation), PS1 with M146L and L286V mutations. These mice accumulate high levels of intra-neuronal Aβ-42 around 1.5 months of age with amyloid deposition around 2 months (27, 28). The adult *Atg5^-/-^* mice were obtained from Herbert W. Virgin from Washington University with permission of Noboru Mizushima (29). All animal experiments were performed according to protocols approved by the Animal Care and Use Committee (IACUC) of the Ohio State University College of Medicine.

### Perfusion of Mice

Mice were anesthetized using ketamine/xylazine mixture and perfused as described before (30). Briefly, heart was surgically exposed, and a perfusion needle was inserted directly into the left ventricle. Perfusion needle was secured in the left ventricle by using a hemostat surgical clamp. An incision was made in the right atrium to create an open circulation. First, heparinized sterile PBS was injected to flush the blood out of the circulation. This was followed by the injection of 4% paraformaldehyde (PFA) fixative solution (30). The brains were dissected and then underwent post-perfusion fixation overnight at 4°C in 4% PFA. Brains were then transferred to 30% sucrose (w/v)-PBS. Brains were embedded in Optimal cutting temperature compound (OCT) and sectioned using a cryostat. Cryosections of 15-20 microns thickness were mounted on glass slides.

### Immunohistochemistry (IHC) for Mouse Tissues

Immunofluorescence (IF) staining of mouse brain sections was performed as previously described (31). Slides were washed 3 times for 15min with PBS to remove residual OCT. The sections were then incubated in the blocking solution (PBS containing 10% donkey serum (cat no: S30-100ml, Millipore Sigma), 2% BSA (Fisher Scientific, BP1600-100) and 0.3% Triton X-100 (Fisher Scientific, BP151-100) for 2h at room temperature (RT). Sections were then transferred to blocking solution containing the primary antibodies (NBR1 (proteintech, 16004-1-AP), IBA1 (Novus Biologicals, NB100-1028), and incubated overnight at 4°C. After that, sections were washed with PBS 3 times for 15min each. Then, they were incubated with the blocking solution containing the secondary antibody Donkey anti-Rabbit IgG (ThermoFisher Scientific, A32790), Donkey anti-Goat IgG (cat. no: A-11058, ThermoFisher Scientific) for 2h at RT. DAPI (Fisher Scientific, D1306) was added on the top of the antibody solution in the last 15min of the incubation period at a final concentration (5ug/ml). Finally, sections were washed with PBS 3 times for 15min. Antifade mounting media (ThermoFisher Scientific, P36934) was added before cover-slipped.

### Confocal Imaging of Mouse Tissue Sections and Imaris Analysis

Fluorescent images were captured on Olympus FV3000 inverted microscope with a motorized stage using 60x/1.4 NA oil objective. Images were taken at z-sections of 0.5μm intervals by using the 488nm, 543nm, and 405nm (for DAPI) lasers. Image reconstructions of z-stacks were generated in Imaris software (Bitplane, Inc.). Volume of NBR1 was analyzed using surface functions and masking tools as described before (32, 33). The measurement of volume was used to evaluate the amount of the target protein in 3D images. Briefly, IBA1 and NBR1 were 3D reconstructed using the Imaris surface function. Surface masking tool was used to create a new channel of NBR1 only confined to the IBA1 positive microglia. Surface function was used for the 3D reconstruction of the microglia NBR1 from the newly created channel. Subsequently, volume statistics were extracted from the 3D reconstructed surfaces. Staining and imaging were performed on 3 brains isolated from 5xFAD mice and 3 brains of age- and sex-matched WT littermates.

### Immunohistochemistry (IHC) and *In Situ* Hybridization (ISH) of Human Tissues

The human brain tissues were equally divided between the hippo- campus and frontal or parietal cortex. All samples were de-identified from the patient’s name, hospital where autopsy was performed, and date of birth (though birth year was maintained). The formalin fixed, paraffin embedded tissues was prepared as per standard operating procedures (entire brain fixed for two weeks, then 1 cm sections embedded in formalin). The tissue was sectioned at DLS: 4-micron sections were placed onto sequentially labeled PLUS slides, baked at 60°C for 30min, then stored in a light tight box at RT. In situ hybridization (ISH) was done using the Leica Bond Max automated platform (Leica, Buffalo Grove, IL) with the modification of substituting the Polyview polymer-peroxidase conjugate from Enzo Life Sciences (Farmingdale, NY) (26). IHC was performed for NBR1, TMEM-19, GFAP and Pyruvate dehydrogenase. The tissues were also tested for Aβ(1-42) after protease digestion (proteinase K solution, Enzo Life Sciences) for 4min at RT. Each antibody was from abcam and used at a dilution of 1:700.

### Human Brain Section Imaging and Analysis

The imaging microscope was from Ventana Biosystems and was a Zeiss Axio Imager M1. Co-expression analyses were done using the Nuance/InForm software (Perkin Elmer) whereby each chromogenic signal is separated, converted to a fluorescence-based signal, then mixed to determine what percentage of cells were expressing the two proteins of interest as previously described. The number of positive cells/200X field was counted with the InForm software or manually. Manual and InForm computer-based counts were found to be equivalent.

### Aβ Peptides and Fibrillization

Aβ peptides in powder form (Anaspec) were initially resuspended in 1% NH_4_OH for 15min to dissolve any pre-formed aggregates per manufacturer instructions, and then diluted to a final concentration of 0.05% NH_4_OH in water prior to fibrillization. Aβ peptides were converted to the fibrillar form of Aβ by incubating monomeric human Aβ(1-42) (cat. no: AS-20276) or human Hilyte Fluor 555 labeled Aβ(1-42) (cat. no: AS-60480-01) at 220μM in water at 37°C for 3days prior to use as previously described (34). Hilyte Fluor is more photostable than the classic fluorescent dyes. They are also highly fluorescent over a broad pH range with little pH sensitivity (Anaspec). Treatment with 1µM Aβ was completed in serum free media for 1h before washing away excess Aβ and adding full media.

### Microglial Isolation and Culture

Microglia were isolated by MACS neural dissociation kit (Miltenyi Biotec, 130-107-677) followed by CD11b magnetic bead (Miltenyi Biotec, 130-093-634) isolation technique to positively select for microglia expressing the pan-microglial marker CD11b as has been described before (35). For experiments evaluating 5xFAD microglia in culture, microglia were isolated from adult mice between 4-6-months-old and age- and sex-matched WT littermate mice. For experiments evaluating WT and *Atg5^-/-^* mice, microglia were isolated from 2-3-month-old mice. Genotypes were age- and sex-matched. For experiments using WT mice, brains from 2 mice were combined as one biologic replicate. For experiments using AD mice, one brain was sufficient per biologic replicate. Experiments with microglia in culture were performed on 3 or 4 unique biologic replicates as indicated. Experiments were performed using both female and male mice. No trends in the data were observed based on sex. Microglia were then cultured on either 24-well plates with pre-coated cover slips with 0.005% Poly-L-lysine (Sigma, P4707) at 2x10^5^ microglia per coverslip. Microglia were cultured in IMDM media (ThermoFisher Scientific, 12440053) supplemented with 10% of heat inactivated fetal bovine serum (FBS) (ThermoFisher Scientific, 16000044), 50% L cell-conditioned media, 0.6x MEM Non-Essential Amino Acids (ThermoFisher Scientific, 11140050) and 0.1% penicillin and streptomycin (Thermo Fisher Scientific, 15140122).

### Fluorescent Aβ(1-42)-555 Degradation Assay and Image Analysis

Primary microglia were plated at 2x10^5^ cells per well on glass coverslips. Two wells from the same biological replicate were treated with 1μM Hilyte Fluor 555 labeled fibrillar Aβ (1-42) for 1h in serum free media. Un-internalized Aβ (1-42) was removed by rinsing with warm full media. One coverslip was fixed with 4% PFA after an additional 2h. The other well was incubated for 48h prior to PFA fixation. Prior to fixation, cells were stained with Hoechst 33342 for 10min (ThermoFisher Scientific, 62249). Images were acquired by confocal microscopy and analyzed for volume of Aβ(1-42)-555 and number of cells by Imaris software. Volume of Fluor Aβ was normalized to the number of cells for each time point (Degradation % = [(Volume Aβ at 3h/cell number – Volume Aβ at 48h/cell number)/(Volume Aβ at 3h/ cell number)]×100). Fluorescent images were captured on Olympus FV3000 inverted microscope with a motorized stage using 10x objective. Images were acquired at z-sections by using 543nm, and 405nm (for DAPI) lasers. Imaris software was used to generate projection images and for analysis. Volume of Fluor Aβ(1-42)-555 was calculated using surface function. Background subtraction function was also used to enhance IF Signal-To-Noise-Ratios (32, 33). Number of cells was counted using spots function in the DAPI channel.

### Down-Regulation of *miR-17* in Primary Microglia

50nM miRNA inhibitor to *miR-17-5p* (antagomir17) (ThermoFisher Scientific, 4464084), or negative inhibitor control (ThermoFisher Scientific, 4464079) were transfected into microglia utilizing the Hiperfect transfection system (Qiagen, 301704) in full media according to the manufacturer’s instructions for 48h prior to performing treating with Aβ.

### LC3 and NBR1 Immunofluorescence of Primary Microglia and Image Analysis

Primary microglia were plated at 2x10^5^ cells per well on glass coverslips overnight. Microglia from 5xFAD mice were treated with antagomir-17 or negative control as described where indicated. The cells were washed once with warm media and then the assigned wells were treated with 1μM Hilyte Fluor 555 labeled fibrillar Aβ (1-42) for 1h prior to washing all the wells 3 times with warm media. The cells were then fixed with 4% PFA either after 2, 4, 6h as indicated for 20min before washing 3 times with PBS. The fixed cells were permeabilized and blocked by incubating in blocking solution (PBS containing 10% donkey serum, 2% BSA and 0.3% Triton X-100) for 2h at RT. They were then transferred to blocking solution containing the primary antibody LC3 (Cell Signaling Technology, 12741) or NBR1 (Proteintech, 16004-1-AP) and incubated overnight at 4°C followed by PBS wash 3 times. The cells were then incubated with the blocking solution containing the secondary antibody goat anti-Rabbit IgG (Thermofisher scientific, A11008) for 2h at RT. Cell nuclei were stained with 5ug/ml DAPI. Antifade mounting media was added before cover-slipped. The Images were taken at z-sections (12 images per cover slip for LC3 and 10 per coverslip for NBR1) of 1μm intervals by using the 488nm, 543nm, and 405nm (for DAPI) lasers. Image reconstructions of z-stacks were generated in Imaris software (Bitplane, Inc.). For LC3 quantification, LC3 volume was analyzed using surface function as described before (32, 33). The measurement of volume was used to evaluate the amount of the target protein in 3D images. For NBR1 positive cells quantification, microglia that express 3 or more NBR1 puncta was manually counted in a blinded manner. Cells were considered positive for NBR1 puncta if they met the selected threshold of 3 puncta. Assigning an appropriate threshold for counting puncta for autophagy studies is discussed in the guidelines for monitoring autophagy report (36).

### Aβ Degradation in HMC3 Microglia

Human microglia cell line (HMC3) was purchased from ATCC (CRL-3304) and cultured in MEM (ThermoFisher scientific, 11995040), supplemented with 10% FBS. Cells were plated at 1x10^5^ cells per well in 12 well plate. Transfection was performed in Opti-MEM media (Gibco, 31985070) with NBR1 sirna (Dharmacon, LQ-062130-01-0005) or its non-targeting negative control next day for 24h before treatment with 1µM fibrillar Aβ (1-42) for 1h. Then, the cells were washed 3 times with PBS, and media was replaced. Cells were lysed at 4 and 24h with TRIzol. Protein extraction and immunoblotting for Aβ were performed to assess the course of Aβ degradation.

### Immunoblot

Protein extraction was performed using TRIzol reagent (Invitrogen Life Technologies, 15596026) according to the manufacturer’s instructions. Briefly, after phase separation using chloroform, 100% ethanol was added to the interphase/phenol- chloroform layer to precipitate genomic DNA. Subsequently, the phenol-ethanol supernatant was mixed with isopropanol to isolate proteins. The Bradford method was used to determine protein concentrations. Equal amounts of protein were separated by 13.5% SDS-PAGE and transferred to a polyvinylidene fluoride (PVDF) membrane. Membranes were incubated overnight with antibodies against mouse NBR1 (Cell Signaling Technology, 9891S), human NBR1 (Proteintech, 16004-1-AP), human beta-amyloid (Cell signaling technology, 8243S), ATG5 (Cell Signaling Technology, 12994S), ATG7 (Cell Signaling Technology, 8558S), Beclin 1 (Cell Signaling Technology, 3495S) and GAPDH (Cell Signaling Technology, 2118). Corresponding secondary antibodies conjugated with horseradish peroxidase and in combination with enhanced chemiluminescence reagent (Amersham, RPN2209) were used to visualize protein bands.

### Quantitative Real-Time PCR (RT-qPCR) for Expression of *MiRs* and Autophagy Genes

Total RNA was isolated from cells that were lysed in TRIzol. Chloroform (cat no: 268320010, Fisher Scientific), isopropanol (cat no: BP2618212, Fisher Scientific), and glycogen (cat no: 10814010, Fisher Scientific) were used to isolate total microglia RNA and its concentration was measured by Nanodrop. The expression of different *MiRs* and autophagy genes was determined as previously described and expressed as relative copy numbers (RCN) (37). Ct values of each gene *or MiR* were subtracted from the average Ct of the internal control. The resulting ΔCt was used in the equation: RCN= (2^-ΔCt^).

### Intracisternal Magna (ICM) Injection of Antagomir-17

To determine the impact of blocking action of *miR-17*, mice received an infusion of antagomir-17 (mirVana miRNA inhibitor; 4464088, Thermofisher) or non-targeting control (4464077) into the cisterna magna (ICM) as described previously (38, 39). Four mice were injected with non-targeting control (3 males and 1 female) and 3 mice were injected with antagomir-17 (2 males and 1 female). The inhibitor and non-targeting control was prepared for *in vivo* ready use by the manufacturer. Mice were briefly anesthetized with isofluorane and the dorsal aspect of the skull was shaved. A 27-gauge needle attached *via* PE50 tubing to a 25 μl Hamilton syringe was inserted into the cisternal magna. To verify entry into the cerebral spinal fluid (CSF), approximately 2 μl of clear CSF was drawn and gently pushed back in and 4 μl of antagomir-17 (dose of 1.2 nmole) was slowly administered and allowed to absorb for 1 min before the needle was removed. Following injection, isofluorane was immediately discontinued and mice were awake, alert and mobile within 3min. Mice were injected once weekly for 4 weeks and then euthanized after 1 more week to isolate microglia by CD11b(+) magnetic bead purification. Microglia were isolated and immediately lysed in TRIzol. ICM injections were utilized as no surgery or cannulae implantation are required, which are inflammatory manipulations, and because substances injected ICM spread readily throughout the central nervous system (CNS) (38).

### Statistical Analysis

All figures display mean and standard error of the mean (SEM) from at least three independent experiments as indicated in the figure legends. Comparisons between groups were conducted with either two-sample t-test, one-way or two-way ANOVA (depending on the data structure) for independent data, but with linear mixed effects models for correlated data to take account of the correlation among observations obtained from the same subject. Holm’s adjustment for multiple comparisons was used as indicated. Adjusted P < 0.05 was considered statistically significant.

## Results

### Microglial Autophagy Is Required for Degradation of Aβ, but Autophagy Proteins Are Poorly Expressed in 5xFAD Microglia

**Figure 1:**
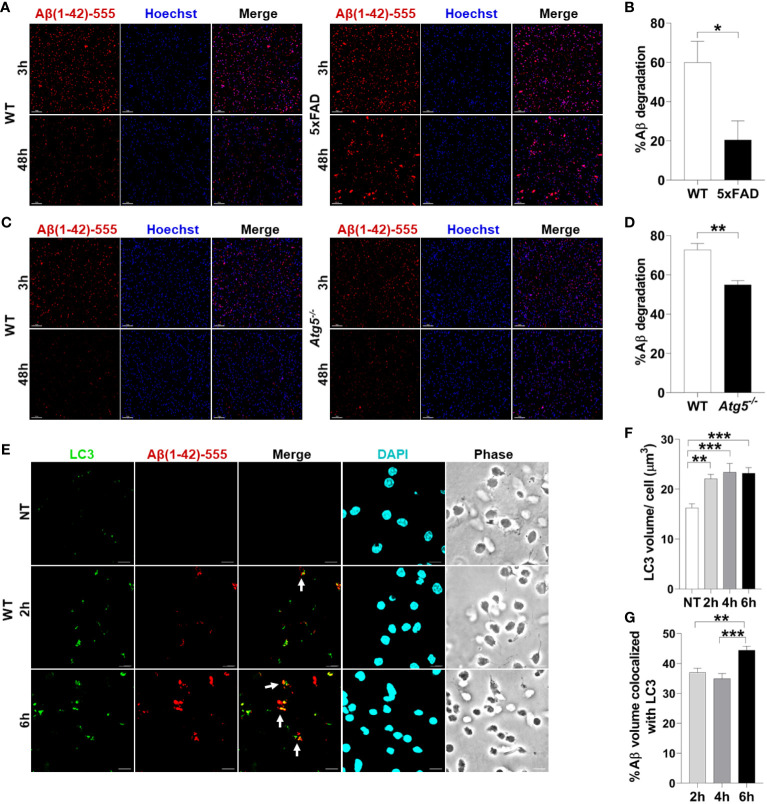
Aβ degradation is diminished in 5xFAD microglia and autophagy knockout microglia. **(A)** Representative confocal images of fluorescent-Aβ (red) in primary microglia isolated from 4-6-month-old 5xFAD and sex- and age-matched WT littermate’s brains treated with Aβ for 3 or 48h. Scale bar: 10µM. **(B)** Quantification of degradation of fluorescent-Aβ (red) by degradation assay of the images represented in **(A)** (n = 3). Values are % of Aβ volume reduction ± SEM calculated by scoring 5 randomly chosen 20x fields of view per biologic replicate. Statistical analysis was performed using unpaired two tailed Student’s t-test. **(C)** Representative confocal images of fluorescent-Aβ (red) added to primary microglia isolated from 2-3-month-old WT and *Atg5^-/-^* mice’ brains for 3 and 48h. Scale bar: 10µM. **(D)** Quantification of images represented in **(C)** were completed as described in **(B)** (n=3). Statistical analysis was performed using unpaired two tailed Student’s t-test. **(E)** Representative confocal images of LC3 (green) in fluorescent-Aβ (red) treated primary WT microglia for 2,6h. Colocalization is represented in yellow as indicated by arrows. **(F)** Quantification of LC3 volume from cells treated as in **(E)** for 2,4,6h performed using Imaris software (n = 4) (12 images per biologic replicate). Statistical analysis performed by matched one-way ANOVA. **(G)** Quantification of LC3 Aβ colocalization from cells treated as in **(E)** for 2,4,6h performed using Imaris software (n = 4) (12 images per biologic replicate). Statistical analysis performed by matched one-way ANOVA. *p ≤ 0.05, **p ≤ 0.01, ***p ≤ 0.001.

There are no studies using primary microglia *in vitro* from adult AD mouse models to determine their ability to degrade Aβ. Therefore, we sought to determine if microglia from 4-6-month-old 5xFAD mice in culture exhibit reduced degradation of fibrillar Aβ, the form most commonly found in plaques (2). The 5xFAD mouse model of AD expresses 5 mutations seen in familial forms of AD under the Thy1 neuronal promoter (27). This model exhibits robust deposition of Aβ, starting at 2-months of age, followed by microgliosis and loss of cognitive function between 4-6 months (27). WT and 5xFAD microglia were isolated by CD11b magnetic bead separation, plated, and then treated with fluorescent Aβ (1µM labeled with acid-resistant Hilyte Fluor 555) for 3 or 48 hours. Aβ degradation (%) was calculated by measuring reduction in fluorescent-Aβ volume between 3 and 48 hours per cell number. We found that microglia cultured from brains of 6-month-old 5xFAD mice are less able to degrade Aβ when compared to their WT counterparts (**Figures 1A, B**).

To determine if autophagy contributes to Aβ degradation and clearance in primary adult microglia, microglia from *Atg5^-/-^* mice were isolated. ATG5 is necessary for elongation of autophagic membranes and enables the recruitment of autophagy molecule LC3. We performed a fluorescent-Aβ degradation assay comparing *Atg5^-/-^* and WT microglia. We found that *Atg5^-/-^* microglia were less capable of Aβ degradation than WT microglia at 48 hours (**Figures 1C, D**). To confirm that primary adult microglia traffic Aβ *via* autophagy, we treated WT microglia with fluorescent-Aβ for 2 and 6 hours and stained for LC3 which is an important marker of autophagosomes. We found that LC3 volume increases upon addition of Aβ (**Figures 1E, F**). Moreover, it colocalizes with Aβ and colocalization increased between 2 and 6 hours (**Figures 1E, G**). Overall, these results indicate that healthy microglia are capable of enclosing Aβ into an autophagosome and degrading it by employing functional autophagy.

**Figure 2:**
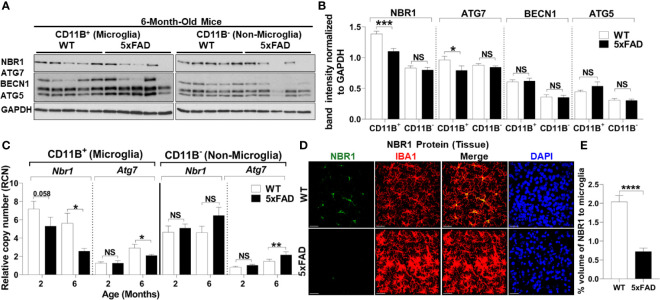
5xFAD microglia exhibit reduced expression of NBR1 and to a lesser extent Atg7 autophagy proteins at 6 months.**(A)** NBR1, ATG7, BECN1, ATG5 and GAPDH immunoblot of primary microglia (CD11b^+^) and non-microglia (CD11b^-^) fractions of 6-month old 5xFAD and sex- and age-matched wild-type (WT) littermate brains (n = 5). **(B)** Densitometry analysis of immunoblots represented in **(A)**. Data represent mean ± SEM (n = 5). Statistical analysis was performed using unpaired two-tailed student’s t-test. **(C)** Relative copy number of *Nbr1* and *Atg7* in primary microglia (CD11b+) or non-microglia (CD11b-) fractions of 2 and 6 month old 5xFAD and sex- and age-matched wild-type (WT) littermate brains (n = 9 for 2 month and n = 12 for 6 month). Statistical analysis was performed using mixed-effect analysis. **(D)** Representative confocal images of NBR1 protein (green) expression on brain tissue sections stained for IBA1+ microglia (red) from 6-month old 5xFAD and age-matched WT littermates. **(E)** Quantification of images represented in **(D)** was analyzed by Imaris software (n = 3). Statistical analysis was performed by unpaired student t-test. *p ≤ 0.05, **p ≤ 0.01, ***p ≤ 0.001, ****p ≤ 0.0001, NS, Not Significant.

To determine the underlying defect responsible for failed autophagy-mediated degradation of Aβ in AD microglia, we determined autophagy protein expression in microglia from 5xFAD mice. Microglia were isolated from 5xFAD mice and age- and sex-matched WT littermates at both 2- and 6-months-old as significant Aβ deposition and microgliosis occurs after 2 months (27). Microglia were purified by magnetic bead positive selection for CD11b and the CD11b-negative fraction was also collected. By immunoblot analysis, expression of autophagy proteins NBR1, ATG7, BECN1, and ATG5 were analyzed in both the microglia and non-microglial fractions (**Figures 2A, B**). NBR1 and ATG7 were significantly less expressed in microglia in 6-month-old 5xFAD mice. This difference was not observed at 2 months, indicating that the expression of autophagy proteins is downregulated in microglia over the course of the disease (**Supplementary Figures 1A, B**). Expression of both *Nbr1* and *Atg7* mRNA was also diminished in 6-month-old (not 2-month old) 5xFAD mice (**Figure 2C**). We did not observe differential expression of ATG5 or BECN1 (**Figures 2A, B**) or any autophagy markers in the microglia fraction of 2-month-old mice (**Supplementary Figures 1A, B**). However, NBR1 and ATG5 were elevated at 2-months in the non-microglia fraction, though the difference disappeared in older mice. The results in **Figures 2A, B** show that the expression of NBR1 is decreased in 5xFAD microglia purified from the brain. Since transcriptional changes may occur during both isolation and culture of microglia (40), we characterized the expression of NBR1 in microglia *in situ*. To do this, NBR1 expression in microglia was analyzed using immunofluorescence staining in 5xFAD hippocampal brain sections from 6-month-old 5xFAD mice. Microglia in 5xFAD mice exhibited significantly lower expression of NBR1 protein (**Figures 2D, E**) compared to WT microglia. Together, these results indicate that degradation of Aβ in 5xFAD microglia is compromised due to diminished expression of autophagy molecules NBR1 and ATG7. Our data also demonstrate that autophagy is necessary for Aβ degradation in microglia.

### Autophagy-Cargo Receptor NBR1 Is Poorly Expressed in Microglia of Human Alzheimer’s Disease in Areas With Aβ-Deposits and Is Necessary for Aβ Degradation by Microglia

**Figure 3:**
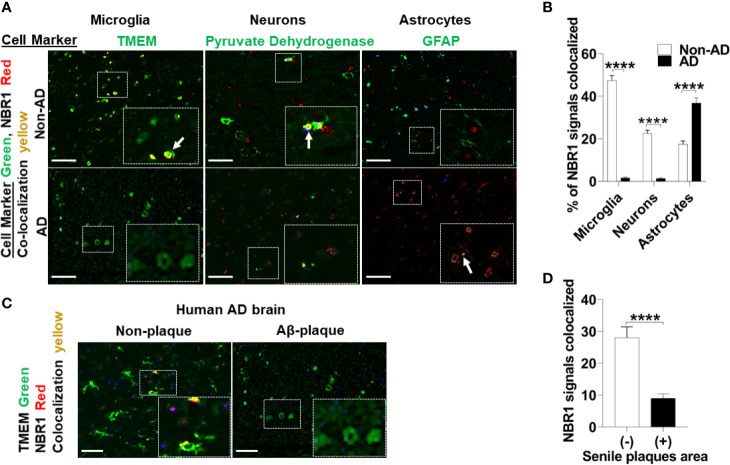
NBR1 is downregulated in microglia of human Alzheimer’s disease mainly in regions with high Aβ burden. **(A)** Co-expression images of Immunohistochemistry of NBR1 (red) expression and cell marker (green) (TMEM for microglia, pyruvate dehydrogenase for neurons and GFAP for astrocytes) in human AD brain tissue sections and age matched no disease control. Colocalization is represented in yellow as indicated by arrows. Scale bar: 100µM. **(B)** Quantification of images represented in **(A)** were performed by Nuance system (CRI) (n = 3) (5 randomly chosen 20x fields of view per biologic replicate were analyzed). Statistical analysis was performed using multiple unpaired two-tailed student’s t-test. **(C)** Representative images of the co-expression analysis of Immunohistochemistry of NBR1 (red) expression and TMEM+ microglia (green) in Aβ deposits negative (-) and positive (+) regions of AD brain tissue sections (n = 3). Scale bar: 100µM. **(D)** Quantification of images represented in **(C)** were performed as described in **(B)**. Statistical analysis was performed using unpaired two-tailed student’s t-test. ****p ≤ 0.0001.

Autophagy linker molecules such as p62 and NBR1 bind to labeled cargo destined for autophagy and recruit LC3 and the nascent autophagosome (15). We hypothesized that NBR1 plays a critical role in autophagic degradation of Aβ, as its expression was diminished in microglia isolated from the brains of 5xFAD mice. To determine if this is the case in AD human subjects, we first examined expression of NBR1 in total human brain tissue homogenates. We obtained snap-frozen brain tissue sections (Brodmann Area 38) from deceased AD patients that were homogenized in TRIzol (characteristics of patients and age- and sex-matched controls can be found in **Supplemental Table 1**). We found that NBR1 protein and mRNA expression were not altered in total brain homogenates from AD human patients compared to no-disease controls (**Supplemental Figures 2A–C**). We hypothesized that NBR1 expression is reduced in specific cell populations so that total brain tissue would obscure these results. We analyzed formalin fixed, paraffin embedded human temporal lobe brain tissue sections from AD and non-AD patients and stained for specific cell populations. Microglia (TMEM+) and neurons (Pyruvate Dehydrogenase+) expressed almost no NBR1 in AD brains in comparison to non-AD which expressed a significant amount of NBR1 (**Figures 3A, B**). In contrast, astrocytes (GFAP+) in AD expressed significantly more NBR1 than in non-AD. We next analyzed regions in the AD brain with intense Aβ deposits build-up, to determine if NBR1 down-regulation is associated with Aβ deposits burden. Microglia in tissue sections with Aβ-deposits had lower expression of NBR1 compared to microglia away from Aβ deposits (**Figures 3C, D**). These results indicate that NBR1 down-regulation is specific to microglia and neurons in AD, particularly in regions with intense Aβ deposition.

**Figure 4:**
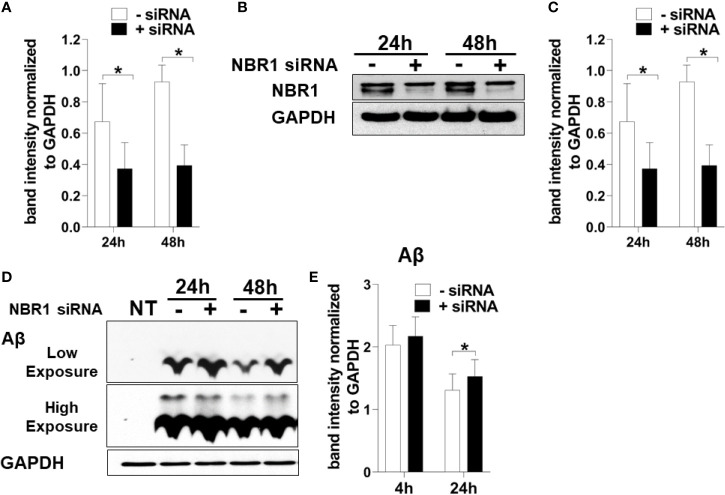
NBR1 is required for Aβ (1-42) degradation. **(A)** *Nbr1* relative copy number in human microglial cell line (HMC3) treated with 50nM *Nbr1* siRNA or non-targeting negative control for 24 and 48h (n = 4). Statistical analysis performed using paired two tailed Student’s t-test. **(B)** NBR1 and GAPDH immunoblot of HMC3 cells treated as in **(A)** (n = 4). **(C)** Densitometry analysis of immunoblots represented in **(B)**. Data represent mean ± SEM (n = 4). Statistical analysis performed using paired two-tailed student’s t-test. **(D)** Immunoblot for Aβ and GAPDH in HMC3 microglia transfected as in **(A)** for 24h and treated with 1 µM of fibrillar Aβ(1-42) for either 4 or 24h to assess the course of Aβ degradation. **(E)** Densitometry analysis of immunoblots represented in **(D)**. Data represent mean ± SEM (n = 4). Statistical analysis was performed using matched two-way ANOVA. *p ≤ 0.05.

To determine if NBR1 is required for Aβ degradation, the human microglia cell line HMC3 was transfected with *NBR1* siRNA or non-target control, leading to successful knockdown of NBR1 at protein and mRNA level (**Figures 4A–C**). We then performed an Aβ degradation assay by immunoblot and discerned that microglia with low NBR1 expression degraded less Aβ at 24 hours when compared to NBR1-expressing microglia (**Figures 4D, E**). These data indicate that NBR1 is necessary for Aβ clearance, however a mechanism behind its low expression in AD microglia remained elusive.

### The Mirc1/Mir17-92 Cluster Is Upregulated in Microglia of Human Alzheimer’s Disease in Areas With Aβ-Deposits

**Figure 5:**
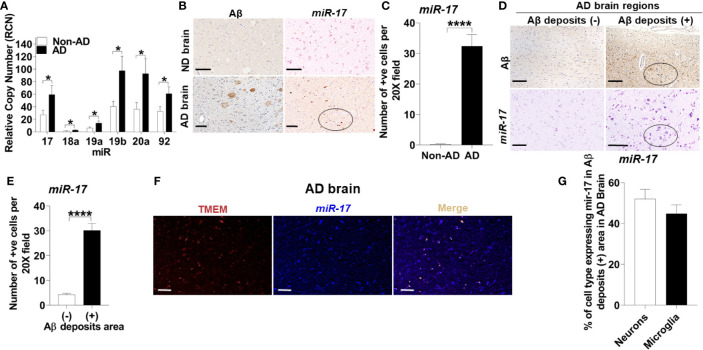
The Mirc1/Mir-17-92 cluster is upregulated in human Alzheimer’s disease and is expressed in microglia. **(A)** Relative copy number of the six miRNAs of the miR-17-92 cluster in human AD brain tissue sections and their age-matched no disease controls (n = 15). Statistical analysis was performed using multiple unpaired two-tailed student’s t-test. **(B)** Representative images of Aβ deposits and in *situ* hybridization of *miR-17* in adjacent brain tissue sections of human AD and no disease control. A region with multiple *miR-17* positive cells is circled. Scale bar: 100µM. **(C)** Quantification of cells expressing *miR-17* represented in **(B)**. Values are mean ± SEM calculated by scoring 3 randomly chosen 20x fields of view (n = 4). Statistical analysis was performed using unpaired two tailed Student’s t-test. **(D)** Representative images of in *situ* hybridization of *miR-17* in Aβ deposits negative (-) and positive (+) area of AD brain tissue sections. Images of Aβ are adjacent tissue sections to corresponding *miR-17* images. A region with multiple *miR-17* positive cells and corresponding Aβ deposits section is circled. Scale bar: 100µM. **(E)** Quantification of images represented in **(D)** as described in **(C)** (N = 4). **(F)** Representative images of the co-expression analysis of in *situ* hybridization of *miR-17* (blue) and Immunohistochemistry of TMEM (as selective marker for microglia) (red) with merge image completed by nuance processing in an Aβ deposits positive area of AD brain tissue sections. Colocalization in the merge image is shown in yellow. Scale bar: 100µM. **(G)** Percentage of the neurons and microglia expressing *miR-17* in Aβ deposits positive area of AD brain tissue sections (n = 3). *p ≤ 0.05, ****p ≤ 0.0001.

To determine the mechanism underlying reduced expression of NBR1, we searched for MicroRNAs that are predicted to target NBR1 and are highly expressed in AD. As members of the Mirc1/Mir17-92 cluster target essential autophagy molecules (24), the expression of the Mirc1/Mir17-92 cluster was evaluated in TRIzol-homogenized brain tissue (Brodmann Area 38) from deceased AD patients. Expression of each member of the cluster, especially *miR-17*, was significantly increased compared to the age-matched non-AD samples (**Figure 5A**). Additionally, the distribution of *miR-17* in fixed brain tissue sections from AD patients and age- and gender-matched controls were analyzed by ISH. Non-AD brains showed low levels of *miR-17* in comparison to AD brains which expressed abundant *miR-17* (**Figures 5B, C**).

To discern the location of highly expressed *miR-17*, we utilized combined ISH and immunohistochemistry (IHC) to determine if the elevation of *miR-17* correlated with the presence of Aβ. We found that the expression of *miR-17* was elevated in areas adjacent to Aβ deposits but not in other sections without deposits from the same AD patient (**Figures 5D, E**).

Microglia are known to robustly proliferate at the site of Aβ deposition (41). Since *miR-17* is highly expressed in regions where Aβ accumulates, we tested if the upregulation of *miR-17* occurs specifically in microglia. By using combined ISH/IHC we evaluated expression of *miR-17* with co-staining for microglia (TMEM+) in AD brain sections. We observed that approximately 45% microglia adjacent to Aβ deposits highly expressed *miR-17* (**Figures 5F, G**). We also analyzed neurons (pyruvate dehydrogenase+) and found that approximately 50% of neurons expressed *miR-17* (**Figure 5G**). Only regions with Aβ deposits in AD brains were analyzed by cell type as both non-AD samples and non-Aβ deposit regions did not express any *miR-17* (**Figures 5B–E**). We concluded that both microglia and neurons in the vicinity of Aβ deposits express *miR-17* in human AD brain. Therefore, *miR-17* may alter expression of specific protein targets in both of these cell types in the AD brain.

**Figure 6:**
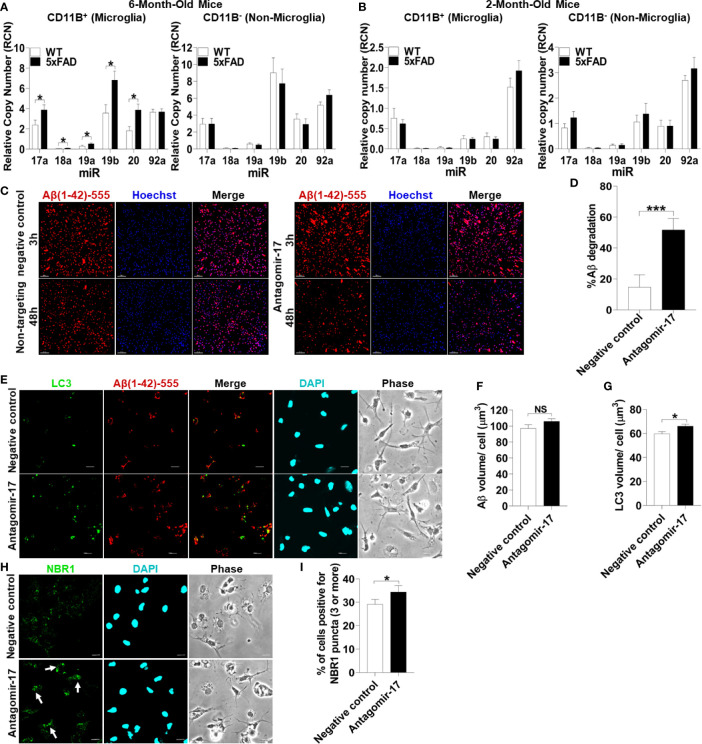
Inhibition of upregulated *miR-17 in* 5xFAD mouse microglia is sufficient to restore Aβ degradation, autophagy, and NBR1 puncta formation. **(A)** Relative copy number of the six miRNAs of the Mirc1/Mir-17-92 cluster in primary microglia (CD11b+) (left panel) and non-microglia (CD11b-) (right panel) fractions of 6-month-old 5xFAD and age-matched wild-type (WT) littermates’ brains (n = 12). Statistical analysis was performed using multiple unpaired two-tailed student’s t-test. **(B)** Relative copy number of the six miRNAs of the cluster in primary microglia (CD11b+)(left panel) and non-microglia (CD11b-) (right panel) fractions of 2 month old 5xFAD and age-matched wild-type (WT) littermates’ brains (n = 9). Statistical analysis was performed using multiple unpaired two-tailed student’s t-test. **(C)** Confocal images of fluorescent-Aβ (red) at 3 and 48h in primary microglia isolated from 5xFAD brains transfected for 48h with either antagomir-17 (50nM) or negative control inhibitor. Scale bar: 10µM. **(D)** Quantification of % degradation of fluorescent Aβ (red) by degradation assay of the images represented in **(A)** (n = 3). Values are % of Aβ volume reduction ± SEM calculated by scoring 5 randomly chosen 20x fields of view. Statistical analysis was performed using paired two tailed Student’s t-test. *p ≤ 0.05, ***p ≤ 0.001. **(E)** Representative confocal images of LC3 (green) in primary microglia isolated from 5xFAD brains treated with 1 µM fluorescent-Aβ (red) for 2h after 48h transfection with either antagomir-17 (50nM) or negative control inhibitor. **(F)** Quantification of Aβ volume in the images represented in **(E)** performed using Imaris software (n = 4) (12 images per biologic replicate). Statistical analysis performed by paired t-test. **(G)** Quantification of LC3 volume from cells treated as in **(E)** performed using Imaris software (n = 4) (12 images per biologic replicate). Statistical analysis performed by paired t-test. **(H)** Representative confocal images of NBR1 (green) in primary microglia isolated from 5xFAD brains treated with 1µM non-fluorescent-Aβ for 2h after 48h transfection with either antagomir-17 (50nM) or negative control inhibitor. Arrows indicate cells with 3 or more discrete NBR1 puncta. **(I)** Quantification of the percentage of cells with 3 or more discrete NBR1 puncta from images represented in **(H)**. Quantification was performed on 10 random fields of view for each condition (n = 3). Statistical analysis performed by paired t-test. NS, Not Significant.

To test if microglia in 5xFAD mice express significant levels of all the members of the Mirc1/Mir17-92 cluster compared to their age-matched WT littermates, microglia were isolated from 6-month-old 5xFAD mice utilizing CD11b magnetic-bead purification. We analyzed both the CD11b (+) microglia fraction and the remaining CD11b (-) non-microglia fraction for the Mirc1/Mir17-92 cluster by RT-qPCR. We found that the members of the cluster are upregulated in isolated microglia, but not in the non-microglia fraction (**Figure 6A**). The non-microglia fraction contains all cell types collected together, such as neurons, and other glia cells including astrocytes, oligodendrocytes and ependymal cells. As different cell types are analyzed together, the elevation of *miR-17* in a specific cell, such as in neurons as seen in human AD (**Figure 5G**), could be masked by other cells that do not express *miR-17*. Since Aβ deposition starts to occur in the 5xFAD mouse after 2 months, we tested if the elevation of the cluster in 5xFAD microglia occurs by the age of 2 months. We purified microglia from 2-month-old 5xFAD mice and WT littermates and found no detectable difference in cluster expression in microglia or non-microglia (**Figure 6B**). Overall, we identified upregulation of the Mir17-92a cluster in both human and mouse AD microglia, which may contribute to dysfunctional autophagy and failed clearance of Aβ.

### Inhibition of Upregulated *miR-17* Is Sufficient to Restore the Ability of 5xFAD Microglia to Degrade Aβ

We hypothesized that treatment with an inhibitor of *miR-17* (antagomir17) would restore the ability of 5xFAD microglia to degrade Aβ *in vitro*. *MiR-17* is highly expressed in AD microglia in human and mouse microglia and is known to target multiple autophagy effectors (24). To detect if 5xFAD microglia in culture lose the differential expression of Mirc1/Mir17-92 cluster, expression of this cluster was analyzed after 24 hours in culture in full media. We found that 5xFAD microglia compared to age-matched littermates still exhibit increased expression of the cluster (**Supplemental Figure 3**). To determine if reducing expression of *miR-17* in AD microglia improves the degradation of Aβ, 6-month-old 5xFAD microglia were transfected with antagomir-17 or non-target negative control. A fluorescent-Aβ degradation assay was performed after 48 hours of antagomir-17 or negative inhibitor transfection. Treatment with antagomir-17 significantly improved Aβ degradation (**Figures 6C, D**). Our data indicate that inhibition of *miR-17* alone is sufficient to significantly improve the degradation of Aβ in 5xFAD microglia *in vitro.* Therefore, targeting all members of the cluster to improve Aβ degradation is not necessary.

To determine if improved Aβ degradation after *miR-17* inhibition is due to recovered autophagy function, 5xFAD microglia were probed for autophagosome marker LC3 after 48 hours of antagomir-17 or negative control transfection. Microglia were treated with Aβ for 2 hours to observe differences in LC3 accumulation after Aβ degradation. Similar amounts of Aβ were associated with microglia treated with antagomir-17 or the negative control after 2 hours (**Figures 6E, F**). Notably, microglia treated with antagomir-17 exhibited increased LC3 volume compared to the negative control, indicating increased presence of autophagosomes (**Figures 6E, G**).

We demonstrated that NBR1 is involved in Aβ degradation and that *miR-17* prevents degradation of Aβ in AD microglia. Importantly, DIANA-TarBase v8 (42), a reference database indexing experimentally supported *MiR* targets, validated NBR1 as a target of *miR-17* (43). Therefore, we quantified NBR1 puncta formation in AD microglia after inhibition of *miR-17*. NBR1 puncta represent receptor clustering which promotes continued autophagosome formation (44). Counting puncta labeled with various autophagy proteins is routinely used in autophagy studies to provide a snapshot of this dynamic process (45). We then examined if AD microglia treated with antagomir-17 would express more NBR1 puncta than the negative control treated microglia. 5xFAD microglia were probed for NBR1 after 48 hours of antagomir-17 or negative control transfection. Microglia were then treated with Aβ for 2 hours to observe differences in NBR1 accumulation before detectable Aβ degradation starts. Cells were considered positive for NBR1 if they met the threshold of 3 puncta per cell. We found that significantly more AD microglia treated with antagomir-17 were positive for NBR1 puncta compared to cells treated with the negative control (**Figures 6H, I**). Taken together, these results indicate that inhibition of *miR-17* improves Aβ degradation, autophagy and NBR1 puncta formation in 5xFAD microglia.

### Targeting *miR-17 In Vivo* Improves the Expression of NBR1

**Figure 7:**
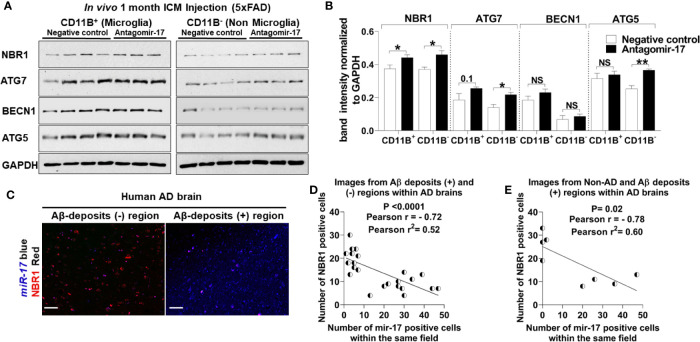
The elevation of miR-17 expression is associated with decreased expression of NBR1 in mouse and human AD brain tissue. **(A)** NBR1, ATG7, BECN1, ATG5 and GAPDH immunoblot of primary microglia (CD11b^+^) (left panel) and non-microglia (CD11b^-^) (right panel) fractions of 2-month-old 5xFAD mice injected in their intracisterna magna (ICM) with 1.2 nmole of either antagomir-17 or non-target negative control once weekly for four weeks. **(B)** Densitometry analysis of immunoblots represented in **(A)**. Data represent mean ± SEM (n = 4 for negative control and 3 for antagomir-17). Statistical analysis was performed using unpaired two-tailed student’s t-test. **(C)** Representative images of the co-expression analysis of Immunohistochemistry of NBR1 (red) expression and in *situ* hybridization of *miR-17* (blue) in Aβ deposits negative (-) and positive (+) area of AD brain tissue sections. Scale bar: 100µM. **(D)** Pearson correlation analysis of *miR-17* and NBR1 expression in AD brains from the images represented in **(C)** (n = 4) (6 wide 20x field images are taken per replicate in which 3 images are taken from Aβ deposits negative and 3 images from Aβ deposits positive). **(E)** Pearson correlation analysis of *miR-17* and NBR1 expression in human brains (n = 8, 4 AD with Aβ deposits and 4 age matched no disease control) (1 wide 20x field image are taken per n). *p ≤ 0.05, **p ≤ 0.01, NS, Not Significant.

**Figure 8:**
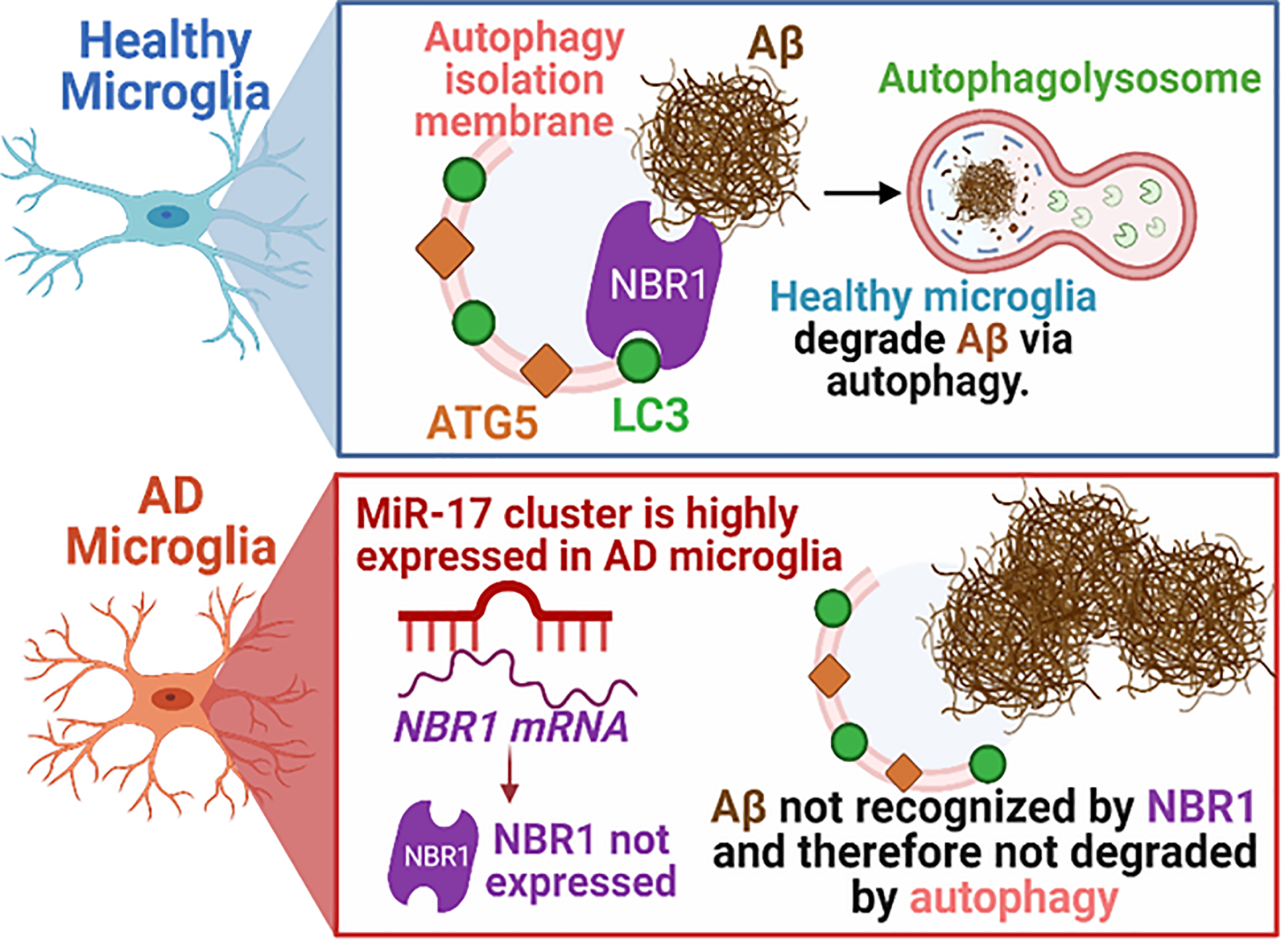
Schematic illustration of the interplay between *miR-17*, NBR1, and Aβ degradation in both healthy and Alzheimer diseased microglia. AD, Alzheimer’s disease; ATG5, Autophagy Protein 5; NBR1, Neighbor of BRCA1 gene 1 Protein; LC3, Microtubule Associated Protein 1 Light Chain 3; Aβ, Amyloid beta; miR-17, microRNA-17. Synopsis: Autophagic clearance of Aβ is disrupted in primary AD mouse microglia (5xFAD). NBR1 is required for Aβ degradation and is downregulated in microglia in human AD in regions with high Aβ burden. The expression of the Mirc1/Mir17-92 microRNA cluster is elevated in AD, including in microglia adjacent to Aβ plaques in human AD and in 5xFAD microglia. Elevated expression of *miR-17* correlates with reduced expression of its predicted target, autophagy receptor NBR1. Inhibiting *miR-17* in primary AD mouse microglia is sufficient to restore Aβ degradation, autophagy, and NBR1 puncta formation in AD microglia *in vitro* and NBR1 expression *in vivo*.

We speculated that reducing the expression of *miR-17* would also bolster the expression of NBR1 *in vivo*. To confirm that antagomir-17 treatment *in vivo* would effectively increase autophagy protein expression in microglia, we injected 5xFAD mice with 1.2 nmole of antagomir-17 once weekly for four weeks by intracisterna magna (ICM) injection. Both the CD11b (+) and CD11b (-) fractions were collected for analysis. Mice treated with antagomir-17 had significantly higher levels of NBR1 in both microglia and the CD11b (-) fraction compared to the non-targeting control (**Figures 7A, B**). We also analyzed expression of ATG7, ATG5 and BECN1 (**Figures 7A, B**). We noted a modest, though non-significant, improvement in ATG7 expression in microglia after antagomir-17 treatment. Additionally, we determined that ATG5 and ATG7 expression were improved in non-microglial cells after antagomir-17 treatment. In order to determine if decreased expression of NBR1 in human AD correlates with increased expression of *miR-17*, a correlation analysis was performed utilizing combined ISH/IHC. First, we performed this analysis in the AD brain in regions with and without Aβ deposits (**Figure 7C**). Decreased expression of NBR1 was highly correlated with increased expression of *miR-17* (**Figure 7D**). Additionally, we completed this analysis on AD brains with deposits together with no-disease controls. Decreased expression of NBR1 was highly correlated with increased expression of *miR-17* (**Figure 7E**). Overall, our results indicate that in AD, the overexpressed *miR-17* correlates with reduced expression of NBR1 which participates in the Aβ degradation in microglia. A schematic illustration of the interplay between *miR-17*, NBR1 and Aβ degradation in both healthy and Alzheimer diseased microglia is suggested in (**Figure 8**).

## Discussion

Healthy microglia play a crucial role in clearing Aβ deposited by neurons in the brain (8). However, microglia become dysfunctional over the course of AD and fail to clear Aβ, and instead they release toxic neuro-inflammatory products (10). Degradation of Aβ can be mediated by microglia by various mechanisms, including release of extracellular enzymes such as neprilysin or insulin-degrading enzyme or by degradation of Aβ following phagocytosis where internalized cargo is delivered to phagolysosomes or autophagolysosomes for degradation (6–8, 12). Previous reports suggested that autophagy machinery degrades Aβ following phagocytosis and that weak autophagy activity in microglia is associated with failed Aβ clearance and unsuccessful regulation of neuro-inflammatory phenotypes (11–13, 18, 46, 47). However, these studies were completed in neonatal microglia or microglia cell lines and did not offer underlying mechanisms for defective autophagy in AD microglia. We demonstrate for the first time using our model of primary adult mouse microglia that Aβ degradation following phagocytosis is dependent on autophagy and that Aβ colocalizes with autophagosome marker LC3. Furthermore, we demonstrate that expression of specific autophagy proteins NBR1 and to a lesser extent ATG7 are diminished in 5xFAD mouse microglia. This is the first study employing adult 5XFAD microglia where we show that NBR1 is required for Aβ degradation. Hence, reduced expression of NBR1 is responsible (at least in part) for failed degradation of Aβ by 5xFAD microglia. This is a very important finding since NBR1 is involved in early steps of autophagy degradation and hence, cannot be remedied by increasing the expression of the downstream autophagy effectors. Moreover, we offer a mechanism underlying dysfunctional autophagy-mediated degradation of Aβ. Our study uncovered that the Mirc1/Mir17-92a cluster is upregulated in 5xFAD mouse and human AD microglia which is associated with decreased expression of autophagy proteins specifically NBR1. Highly expressed *miR-17* in AD microglia targets NBR1 leading to its downregulation which renders AD microglia unable to degrade Aβ by the autophagy machinery. We found that inhibition of *miR-17* alone improved degradation of Aβ, which offers a unique drug target.

*MiRs* are heavily implicated in both AD pathogenesis (21, 23) and autophagy (20), but there was a lack of evidence linking these two processes. Members of the Mirc1/Mir17-92 cluster negatively target expression of autophagy proteins, leading to weak autophagy activity in different disease conditions including multiple cancers and cystic fibrosis (24, 48). We discovered that the Mirc1/Mir17-92a cluster is upregulated in microglia in the temporal lobe of human AD patients compared to sex- and age-matched patients with no known dementia. These findings are distinct from previous studies describing downregulated expression of the Mirc1/Mir17-92 cluster during aging and senescence using total brain homogenates (25).

We observe elevated expression of *miR-17* in microglia and neurons in human AD adjacent to Aβ deposits. Several reports suggest that dysfunctional autophagy in AD neuronal cells contributes to the over-production of abnormal Aβ and subsequent aggregation which instigates the process that leads to AD pathology (49). We also demonstrate that the Mirc1/Mir17-92a cluster is significantly upregulated in 6-month-old 5xFAD mice, which is after the detection of deposition of Aβ plaques in this model (27). Therefore, the dysregulation of Aβ deposition from neuronal cells precedes autophagy dysfunction in surrounding microglia. It is possible that long-term exposure to Aβ plaques provokes the upregulation of *miR-17* in surrounding cells. Yet, in our hands, exposure of non-diseased microglia to Aβ for several days *in vitro* did not increase the expression of *miR-17* (data not shown). Alternatively, the Mirc1/Mir17-92 cluster is consistently upregulated in highly inflammatory diseases including tumor sites and in the lungs of cystic fibrosis patients especially during pulmonary exacerbations (50). Therefore, it seems that the deposition of Aβ plaques in the AD brain instigates micro-environmental effects that collectively lead to the upregulation of the cluster and disruption of autophagy in microglia, which requires further investigation. Notably, we report reduced microglial expression of NBR1 and increased microglial expression of *miR-17* in 6-month-old 5xFAD mice but not in 2-month-old 5xFAD mice. It is possible that this delayed autophagy inhibition is a response to the gradual deposition of Aβ in this model after 2-months or due to other consequences downstream of Aβ deposition and AD pathobiology progression. On the other hand, it is possible that other factors gradually augment the expression of *miR-17* in microglia which in turn perturbs autophagy and leads to the accumulation of Aβ. Previous analyses of *MiR* expression in AD whole brain homogenates have not detected the upregulation of members of the cluster (51, 52). Our approach demonstrates that the expression of *miR-17* is specific to brain region, Aβ pathology and cell type, which may account for our discovery. Thus, it necessary to analyze individual cells instead of total homogenates where such findings may be missed.

Cargo that is destined for autophagy is selected for degradation after recognition by specific receptors, such as SQSTM1/p62, Optineurin or NBR1. Cho and colleagues demonstrated that Optineurin interacts with Aβ and is required for its degradation (12). However, Optineurin and p62 are highly expressed in the AD brain (12). In contrast, we demonstrate that the expression of NBR1 is decreased in human AD microglia present adjacent to Aβ deposits and is inversely correlated with expression of *miR-17*. The absence of NBR1 in AD microglia can explain the reduced degradation of Aβ. This is corroborated by the fact that in HMC3 microglia treated with siRNA to NBR1, Aβ degradation is significantly reduced. Importantly, p62 and Optineurin, despite their abundance in AD brains, did not substitute NBR1 and mediate Aβ degradation. It has been proposed that these receptors have redundant functions, however emerging reports in different fields demonstrate that it is not always the case (14). Hence, an approach that will increase the expression of other downstream autophagy effectors without correcting NBR1 would fail to improve Aβ degradation in AD microglia.

Notably, we do not rule out that autophagy proteins may also play a role in phagocytosis as demonstrated by other groups (46, 47). An elegant study from Heckman and colleagues characterized a non-canonical role for autophagy marker LC3 in the uptake of Aβ and receptor recycling (47). This study did not observe a contribution of autophagy to degradation of Aβ in a microglia cell line at 24 hours. This does not contradict our findings since we used primary adult microglia and we observed a significant difference in degradation of Aβ at 48 hours when comparing WT to 5xFAD or *Atg5^-/-^* microglia. Our result indicates a role for autophagy during Aβ degradation distinct from any dysfunction in internalization. Additionally, autophagy has other critical functions in microglia and other immune cells, including regulation of inflammation (12, 18). As *miR-17* inhibits the expression of multiple autophagy proteins, it may also impact the inflammatory response of AD microglia.

The inhibition of *miR-17* improved degradation of fluorescent-Aβ in primary AD microglia, and hence high expression of *miR-17* inhibits degradation. This is the first report demonstrating that the expression of a *MiR* can alter degradation function in AD microglia. We and others reported that *miR-17* has multiple proposed autophagy targets, including *Atg5*, *Beclin-1*, *Atg7*, *Atg12*, *Atg16L1* and *Nbr1* in other cell types (24, 42, 43). We found that inhibition of *miR-17* improved expression of NBR1 in microglia *in vivo*, which we propose leads to improved autophagic degradation of Aβ. Further, we demonstrated that inhibiting *miR-17* in primary 5xFAD microglia increased expression of LC3 and accumulation of NBR1 puncta after treatment with Aβ. LC3 accumulation reflects increased presence of autophagosomes, indicating that reducing *miR-17* improved both Aβ degradation and autophagy function. NBR1 puncta are indicative of clustering of the NBR1 receptor, which can then promote continued autophagosome formation (44). These data indicate that *miR-17* is a potential therapeutic target in AD which could recover functional autophagy in microglia thus enabling improved Aβ clearance and improved cognition.

Several challenges may arise in seeking to measure cognition and Aβ clearance in 5xFAD mice. Our *in vivo* work was performed over the course of one month in a small cohort of young (2-month-old) mice. Robust differences in cognition are not observed in 5xFAD mice until about 5 months (27). Therefore, optimizing a timeline for several months of injections in a large cohort of mice will be critical. ICM injection resulted in improved expression of NBR1, ATG5 and ATG7 in non-microglial cells in our study. It is possible that inhibiting *miR-17* in all cell types may be beneficial as it could promote functional autophagy in neurons. To the best of our knowledge, the role of NBR1 and the Mirc1/Mir17-92 cluster in neurons in AD has not been explored warranting further mechanistic investigation. Neurons adjacent to Aβ-plaques express more *miR-17* and less NBR1 in human AD brain tissue. If overexpressed *miR-17* is found to be detrimental to autophagy function in neurons as well, it would be even more critical to find approaches to control *miR-17* expression as a therapeutic option in AD. Of note, this cluster of *MiRs* has many other biological functions and potential roles in AD, including that *miR-17* is shown to alter expression of the amyloid precursor protein (APP) (53). Therefore, it would be important to restore normal levels of the *MiR*, rather than completely inhibiting its expression. Alternatively, microglia-targeted therapies may be needed. This also highlights the importance of focusing on a specific *MiR* target for thorough mechanistic and therapeutic evaluation, rather than inhibiting the entire cluster. Even though other members of the Mirc1/Mir17-92a cluster are known to target autophagy proteins (24) and could similarly inhibit efficient Aβ degradation, targeting the entire cluster would also increase the number of off-target effects. Interestingly, we found increased levels of NBR1 in astrocytes in human AD. Weak autophagy activity in astrocytes expressing AD risk variant ApoE is linked to decreased clearance of Aβ (54). This points to the possibility that unique mechanisms of autophagy dysregulation exist within different cell types within the same region in the same organ. As the field continues to explore therapeutic options for stimulating autophagy in AD, it will be vital to identify these unique mechanisms and ensure that we employ complementary approaches that improve autophagy function in designated cell types.

Taken together, our results indicate that autophagic clearance of Aβ by AD microglia is defective due to *miR-17*-mediated autophagy inhibition and reduced expression of NBR1. Improving expression of autophagy proteins by inhibiting the elevated *miR-17* improved the degradation of Aβ *in vitro* in microglia. Therefore, our data indicate *miR-17* is a potential therapeutic target to improve autophagic clearance of Aβ in AD microglia.

## Data Availability Statement

The raw data supporting the conclusions of this article will be made available by the authors, without undue reservation.

## Ethics Statement

De-identified human samples were obtained in agreement with the NIH Neurobiobank or the Discovery Life Science Biobank. Written informed consent for participation was not required for this study in accordance with the national legislation and institutional requirements. The animal study was reviewed and approved by Animal Care and Use Committee (IACUC) of the Ohio State University College of Medicine.

## Author Contributions

Conceptualization, AA, SE, KD, and ME. Formal analysis, SE, KD, and ME. Investigation, SE, KD, ME, ND, MA, KK, AB, KH, CC, AH, AAK, HK, SN, HA, XZ, EC-B, HH, SS, AM, GN, RB, MG, and AA. Resources, AA. Writing – original draft, SE, KD, and ME. Writing – review & editing, SE, KD, ME, ND, MA, KK, AB, KH, CC, AH, AAK, HK, SN, HA, XZ, EC-B, HH, SS, AM, GN, RB, MG, and AA. Project administration, AA, SE, KD, and ME. Supervision, AA. Funding acquisition, AA. All authors contributed to the article and approved the submitted version.

## Funding

SE and AB were supported by funding from the Egyptian ministry of higher education. KH was supported by a Cure Cystic Fibrosis Columbus training grant and the National Institutes of Health T32 Infectious Disease Institute (OSU) training grant. KK was supported by Deutsche Forschungsgemeinschaft (DFG - German Research Foundation) and a Cystic Fibrosis Foundation Postdoctoral research grant. AA was supported by Taawon Welfare Association, West Bank, Palestine and Bank of Palestine. GN was supported by a grant from the Alzheimer’s Drug Discovery Foundation (ADDF) (grant # 20160204). Studies in the Amer laboratory are supported by Ohio State University College of Medicine DFA funds and NIAID R01 AI24121 (AD supplement), NHLBI R01 HL127651-01A1, R21AG067755-01A1, R21AG064899-01A1.

## Conflict of Interest

AM and GN were employed by Gnome Diagnostic.

The remaining authors declare that the research was conducted in the absence of any commercial or financial relationships that could be construed as a potential conflict of interest.

## Publisher’s Note

All claims expressed in this article are solely those of the authors and do not necessarily represent those of their affiliated organizations, or those of the publisher, the editors and the reviewers. Any product that may be evaluated in this article, or claim that may be made by its manufacturer, is not guaranteed or endorsed by the publisher.
