# Supplementary figures and images for: Elevated Expression of MiR-17 in Microglia of Alzheimer’s Disease Patients Abrogates Autophagy-Mediated Amyloid-β Degradation
Source: Front Immunol. 2021 Jul 27;12:705581. doi: 10.3389/fimmu.2021.705581 (PMC8379081; doi:10.3389/fimmu.2021.705581)

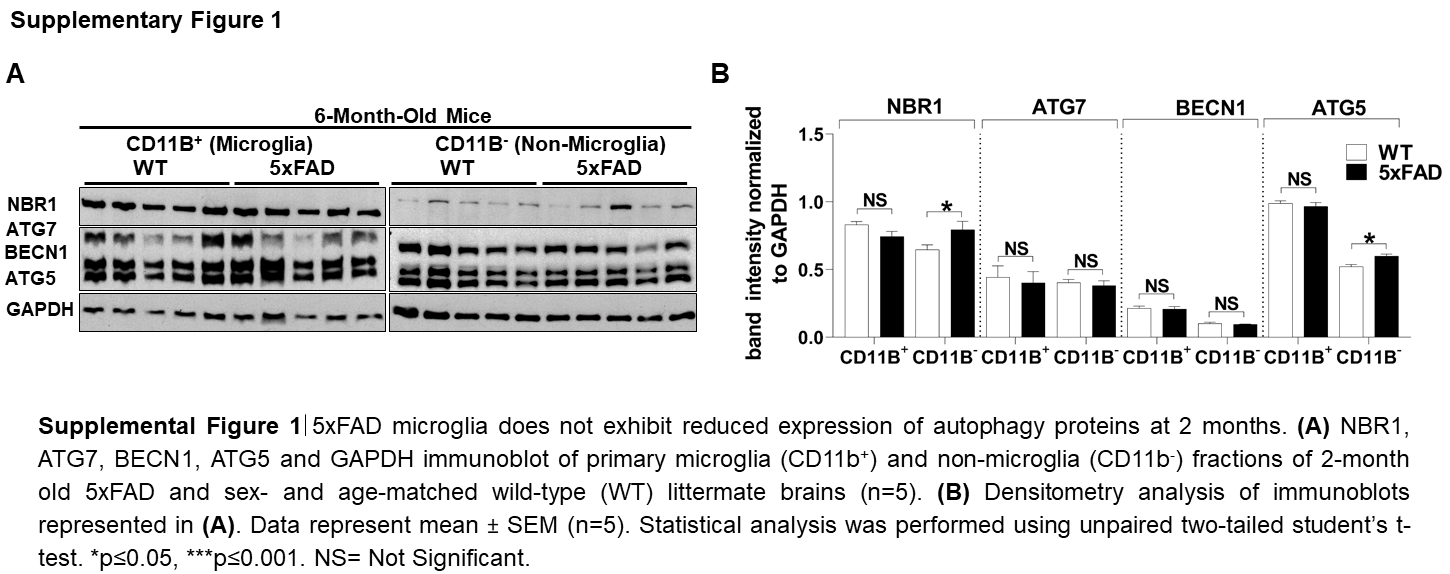

Supplement: Supplementary file 1 [file Image_1.tif]

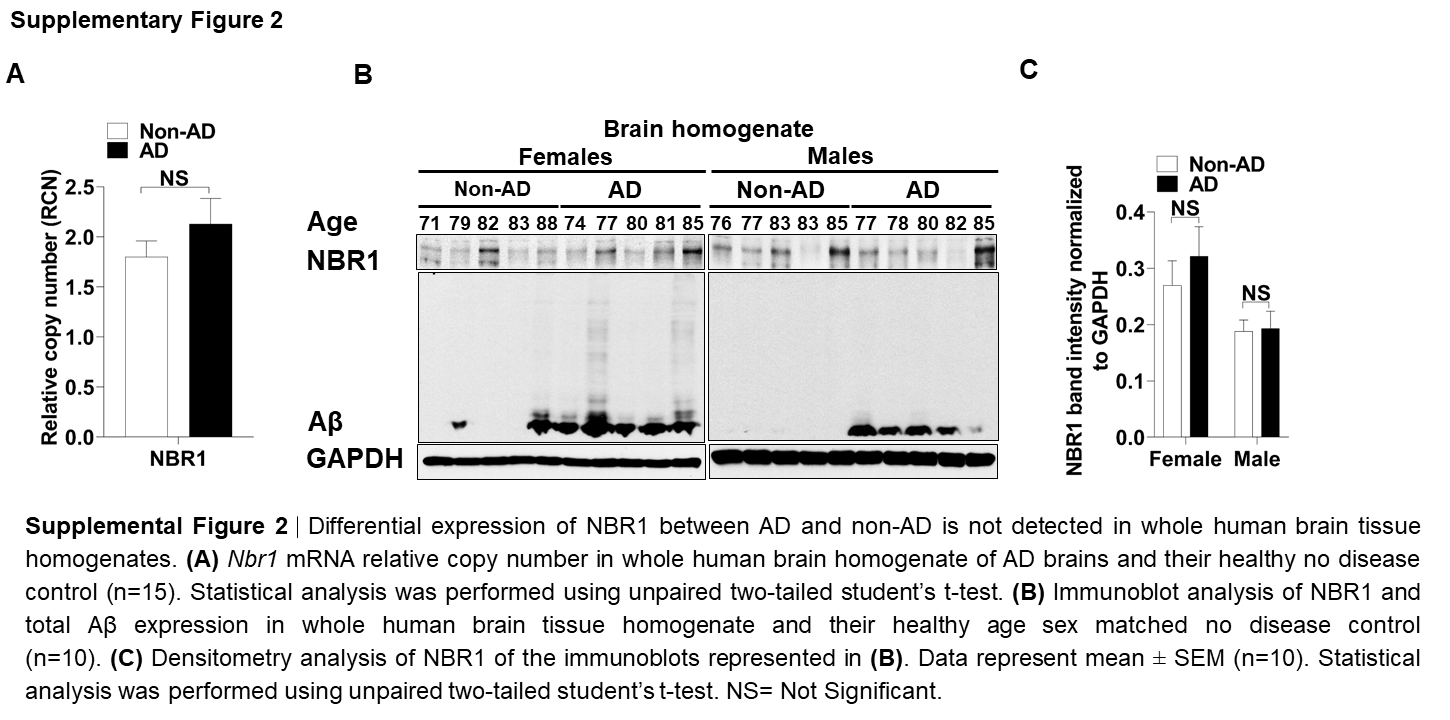

Supplement: Supplementary file 2 [file Image_2.tif]

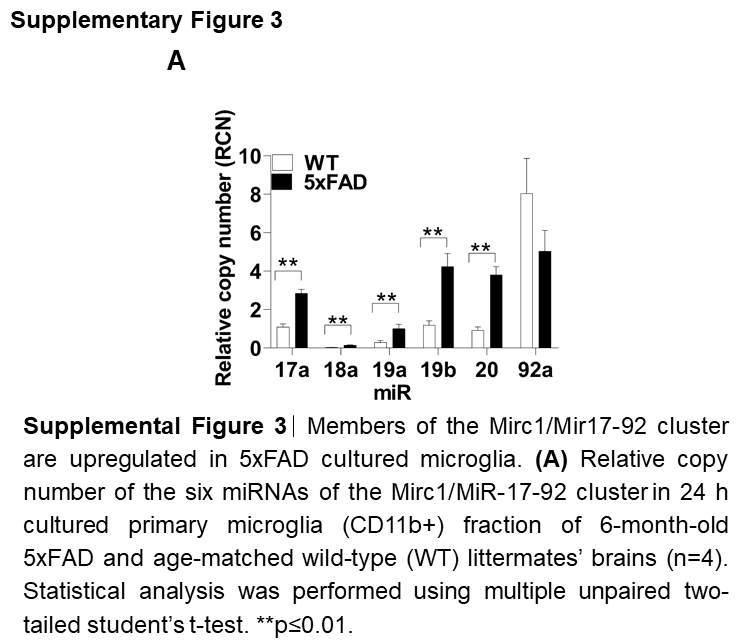

Supplement: Supplementary file 3 [file Image_3.tif]
