# Supplementary material for: Elevated Expression of MiR-17 in Microglia of Alzheimer’s Disease Patients Abrogates Autophagy-Mediated Amyloid-β Degradation
Source: Front Immunol. 2021 Jul 27;12:705581. doi: 10.3389/fimmu.2021.705581 (PMC8379081; doi:10.3389/fimmu.2021.705581)
Supplement: Supplementary file 4 [file Table_1.pdf]

Supplementary Information:

Supplemental Table 1│The patient demographic and clinical characteristics of the human samples used in the study. Y: years, BA: Brodmann's area, h:hours, M:male, F:female, W:White, H:Hispanic, AA:African American.

Brain Endowment Bank Tissue order #899 (Temporal Pole (BA38))						
	Tissue Code	Brain Type	Sex	Age (y)	Race	Autolysis (h)
1	HBDM_001_18_BA38	Alzheimer's disease (A2,B3,C3)	M	60	W	36.5
4	HBFQ_001_18_BA38		M	77	W	18
5	HBPK_001_18_BA38		M	78	W	24
2	HBDA_001_18_BA38		M	80	W	22.13
3	HBIB_001_18_BA38		M	82	W	11.75
6	HBFF_001_18_BA38		M	85	W	17.6
13	HBIG_001_18_BA38		F	60	W	7.91
7	HBIH_001_18_BA38		F	62	W	3.66
8	HBIQ_001_18_BA38		F	66	W	17.66
14	HBFR_001_18_BA38		F	69	W	22
10	HBGU_001_18_BA38		F	74	W	26.5
11	HBEK_001_18_BA38		F	77	W	25.3
12	HctYN_001_18_BA38		F	80	W	6.5
9	HBLZ_001_18_BA38		F	81	W	11.2
15	HBDI_001_18_BA38		F	85	W	7.92
17	Hct15HAL_001_18_BA38	Non-dementia control	M	61	H	12.5
16	HctYP_0001_18_BA38		M	75	W	14.16
21	HctZR_001_18_BA38		M	76	H	27.87
20	HctZP_001_18_BA38		M	77	W	14.72
22	Hct15HBC_001_18_BA38		M	83	H	25
18	HBDE_001_18_BA38		M	83	H	29.55
19	HctZZT_001_18_BA38		M	85	W	15.5
26	HctYB_001_18_BA38		F	60	AA	27.35
23	HctZD_001_18_BA38		F	62	W	18.9
29	HctZL_001_18_BA38		F	65	H	27
25	HctYH_001_18_BA38		F	71	H	16.4
27	HctZA_001_18_BA38		F	79	W	17.8
24	HctZZC_001_18_BA38		F	82	W	14.2
28	Hct15HBF_001_18_BA38		F	83	W	6
30	HctZZG_001_18_BA38		F	88	W	18.1
